# *Helicobacter pylori*, Atherosclerosis, and Coronary Artery Disease: A Narrative Review

**DOI:** 10.3390/medicina61020346

**Published:** 2025-02-16

**Authors:** Angela Saviano, Maria Rita Morabito Loprete, Giulia Pignataro, Andrea Piccioni, Antonio Gasbarrini, Francesco Franceschi, Marcello Candelli

**Affiliations:** 1 Emergency, Anesthesiological and Reanimation Sciences Department, Fondazione Policlinico Universitario A. Gemelli—IRCCS of Rome, 00168 Rome, Italy; angela.saviano@policlinicogemelli.it (A.S.); maria.morabitoloprete01@icatt.it (M.R.M.L.); giulia.pignataro@policlinicogemelli.it (G.P.); andrea.piccioni@policlinicogemelli.it (A.P.); francesco.franceschi@policlinicogemelli.it (F.F.); 2 Medical and Surgical Science Department, Fondazione Policlinico Universitario A. Gemelli—IRCCS of Rome, 00168 Rome, Italy; antonio.gasbarrini@policlinicogemelli.it

**Keywords:** *Helicobacter pylori*, CAD, infection, stroke, CagA

## Abstract

Coronary artery disease (CAD) is one of the leading causes of death worldwide, significantly contributing to mortality in both developed and developing nations. CAD arises from a combination of risk factors, including atherosclerosis, dyslipidemia, hypertension, diabetes, and smoking. In recent years, growing evidence has suggested a potential link between infectious agents and cardiovascular diseases. Among these, *Helicobacter pylori* (*H. pylori*) infection has been hypothesized for over a decade to play a role in the pathogenesis of CAD. This hypothesis is based on the bacterium’s ability to trigger host inflammatory or autoimmune responses, potentially contributing to the progression of atherosclerotic plaques and coronary events. The association between *H. pylori* infection and CAD is of considerable interest as it opens new avenues for prevention and management strategies in cardiovascular health. Understanding this relationship could lead to innovative approaches to reducing the burden of CAD, particularly in populations with a high prevalence of *H. pylori*. In this review, we aim to provide a comprehensive overview of the most recent evidence on the involvement of *H. pylori* in the development and prognosis of CAD. By analyzing and synthesizing current findings, we seek to shed light on unresolved questions and clarify the ambiguous aspects of this potential connection. Our goal is to contribute to a deeper understanding of how *H. pylori*, may influence cardiovascular disease and to inspire further research in this critical area.

## 1. Introduction

*Helicobacter pylori* (*H. pylori*) is a gram-negative, acidophilic, spiral-shaped bacterium that primarily colonizes the stomach and duodenum. It is a major cause of acute gastritis, one of the most prevalent infections globally, affecting a significant portion of the population. In addition to acute gastritis, it is implicated in chronic gastritis, peptic ulcer disease, gastric adenocarcinoma, and gastric mucosa-associated lymphoid tissue (MALT) lymphoma [1]. It is estimated that in developing countries, between 70% and 90% of the population is infected with *H. pylori* [2]. The bacterium can be transmitted through both oral–oral and fecal–oral routes, either directly from person to person or indirectly through contaminated surroundings [3]. *H. pylori* is capable of colonizing and persisting in the gastric lumen. The expression of urease activity and flagellar motility are essential for its survival and function, allowing *H. pylori* to penetrate the mucus layer of the stomach [4]. Moreover, flagella and adhesins such as SabA, BabA, and HopQ facilitate colonization and promote the formation of biofilms, which are aggregates of microorganisms within a hydrated matrix of extracellular substances that protect *H. pylori* against antibiotics and harsh environments [5]. Several risk factors for infection include dietary habits, smoking, water contamination [6], and gut microbiota [7]. *H. pylori* infection can be diagnosed using both non-invasive and invasive methods. Non-invasive methods include the detection of *H. pylori* antigens in stool samples, the urea breath test (UBT), or the detection of antibodies in serum, urine, and oral samples [8]. Invasive tests include histopathology, biopsy cultures, rapid urease tests, and fluorescent in situ hybridization [8]. Treatment of *H. pylori* infection typically involves a combination of antibiotics and proton pump inhibitors. Treatment regimens are tailored based on factors such as the patient’s age, symptoms, concomitant medications, local antibiotic resistance patterns, treatment availability, and associated costs [9]. As previously mentioned, *H. pylori* has been associated with gastrointestinal conditions (such as acute and chronic gastritis, peptic ulcers, and cancer) but it is involved in the pathogenesis of various extra-gastric conditions. These include idiopathic iron deficiency anemia, vitamin B12 deficiency, immune thrombocytopenic purpura (ITP), neurodegenerative diseases such as Alzheimer’s and Parkinson’s disease, an increased risk of preeclampsia in infected women, and cardiovascular diseases [10,11,12]. Several studies have reported an association between *H. pylori* and coronary artery disease (CAD), though the relationship remains a topic of debate. The prevalence of CAD varies significantly across different geographical regions, ethnicities, and genders, but it remains one of the leading diseases affecting the global population. Risk factors for the development of CAD include lifestyle choices, environmental influences, and genetic predispositions. The widespread prevalence of these risk factors in otherwise healthy individuals highlights the potential for an increased incidence of CAD shortly [13]. Numerous projects have been conducted to assess the incidence of cardiovascular diseases across various populations. The World Health Organization (WHO) has coordinated the “MONICA (Monitoring Trends and Determinants in Cardiovascular Diseases) Project” which consists of a multicenter international collaborative study to measure the risk factors (cigarette smoking, blood pressure, and serum lipids and cholesterol) and determinants of cardiovascular diseases (coronary heart attacks and strokes), over 10 years. The population included women and men aged 25–64 years. About 39 collaborating centers from 26 countries in North America, Europe, and the Western Pacific are collaborating in this project, using a standardized protocol and covering a population of about 10 million to identify trends in mortality and morbidity for cardiovascular diseases in defined communities in different countries and to measure how these trends are related to changes in both risk factor and/or medical care [14]. Whereas the INTERHEART study provided valuable insights into the prevalence of CAD across diverse populations. The INTERHEART study was a case-control study conducted in 52 countries, (15,152 cases, 14,820 controls) to evaluate the effect of potentially modifiable risk factors associated with myocardial infarction. Both in women and in men, and for all ages in all regions, the authors reported an association with risk of coronary heart disease and hypertension, diabetes, abdominal obesity, smoking, abnormal cholesterol, alcohol use, consumption of fruits, and vegetables, and regular physical activity. The authors measured the odds ratio (OR) and population-attributable risks (PAR). They found for hypertension (OR 1.91, PAR 17.9%), diabetes (OR 2.37, PAR 9.9%), smoking (OR 2.87 for current vs. never, PAR 35.7% for current vs. never), alcohol consumption (OR 0.91, PAR 6.7%), daily consumption of fruits and vegetables (OR 0.70, PAR 13.7% for lack of daily consumption), regular physical activity (OR 0.86, PAR 12.2%) that prevention can be based on similar principles almost worldwide [15]. In 2016, the American Heart Association published an updated report on heart disease and stroke statistics, revealing that CAD affects 15.5 million individuals over the age of 20 in the United States. This prevalence was found to be increasing with age in both men and women [16]. Intensive epidemiological research has linked CAD to risk factors such as smoking, diabetes, hyperlipidemia, and hypertension [17,18,19,20]. Interestingly, a growing number of studies have demonstrated a link between CAD and various infectious agents, including *H. pylori*, *Chlamydia pneumoniae*, and cytomegalovirus. For example, Eeskandarian et al. [21], showed in a prospective study, the effects of *H. pylori* on the incidence of cardiovascular events in 433 patients presenting with acute coronary syndrome (ACS). The study’s key finding was a positive association between *H. pylori* seropositivity and the incidence of short-term adverse cardiovascular events within the first month after an ACS episode. This review aims to summarize the most important and recent research linking *H. pylori* infection and CAD.

## 2. Pathophysiology

Several studies have explored the mechanisms by which *H. pylori* may contribute to the development of atherothrombosis, including chronic inflammation and direct injury to the vessel wall. These processes can promote the progression or rupture of atherosclerotic plaques, as well as trigger systemic inflammation, both of which are linked to *H. pylori* colonization of the stomach [21]. These inflammatory processes can induce prothrombotic changes in the blood, affecting both plasma (e.g., hyperfibrinogenemia and altered coagulation) and platelets (e.g., increased platelet count, activation, and aggregation), thereby contributing to the development of acute coronary syndrome (ACS) [21,22]. It has been demonstrated that *H. pylori* can stimulate inflammatory cells and trigger the excessive production of cytokines within atherosclerotic plaques, leading to local endothelial and vascular dysfunction. *H. pylori* infection also induces inflammatory mediators such as interleukin-1 (IL-1), interleukin-6 (IL-6), C-reactive protein (CRP), and tumor necrosis factor-alpha (TNF-α), all of which contribute to plaque instability [23]. In addition, *H. pylori* can enter endothelial cells via exosomes containing the cytotoxin-associated gene A (CagA), which causes endothelial damage. The bacterium also secretes another virulence factor, vacuolating cytotoxin A (VacA), which reduces nitric oxide (NO) levels, thereby impairing endothelial function [24,25]. The expression of P-selectin increases following *H. pylori* infection, and the interaction between von Willebrand factor (vWF), released by platelets, and P-selectin promotes platelet aggregation, elevating the risk of thrombosis and, consequently, increasing the risk of CAD [26]. It should also be mentioned that *H. pylori* infection can exacerbate conditions such as hypertension, dyslipidemia, hyper-homocysteinemia, diabetes, and impaired glucose tolerance, all of which are established risk factors for cardiovascular disease [27]. A recent meta-analysis confirmed that *H. pylori* infection was significantly associated with arterial hypertension [28]. Additionally, it has been shown that the eradication of *H. pylori* in patients with essential hypertension can reduce blood pressure values, particularly diastolic pressure [29]. Izhari et al. [30] reported that patients infected with *H. pylori* had a higher risk of elevated serum levels of total cholesterol, triglycerides, and low-density lipoprotein (LDL) cholesterol, as well as reduced levels of high-density lipoprotein (HDL) cholesterol. This effect on lipids could be attributed to a reduction in the activity of paraoxonase and arylesterase, along with an increase in lipid hydroperoxide and total thiol (SH) levels [30]. The interaction between *H. pylori* infection and diabetes has been extensively studied. Evidence suggests that diabetic patients exhibit poorer glycemic control, which in turn serves as a risk factor for the development of CAD [31]. Finally, *H. pylori* infection has been associated with low serum levels of vitamin B12 and folic acid, which consequently lead to hyper-homocysteinemia. Elevated homocysteine levels are a significant factor in the development of atherosclerosis due to their harmful effects on endothelial cells, promoting the formation of atherosclerotic plaques and contributing to vascular diseases [32,33,34].

### 2.1. Cytotoxin-Associated Gene Antigen and Atherosclerosis

**Figure 1:**
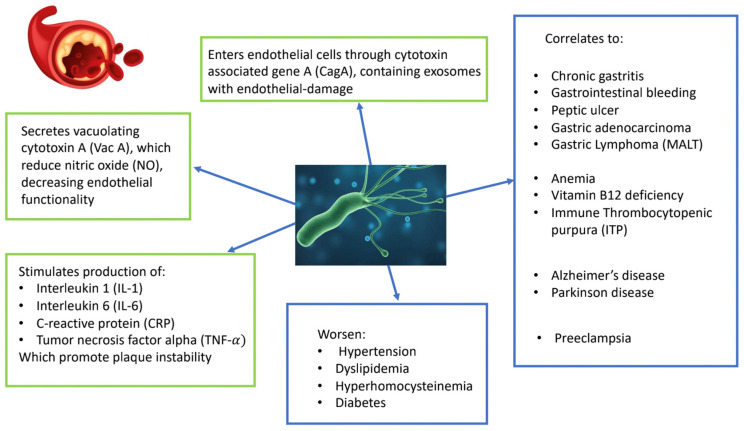
*H. pylori*-related pathophysiological mechanisms associated with coronary artery disease.

The CagA protein is a virulence factor produced by *H. pylori.* It is encoded by the CagA gene, which is part of the cag pathogenicity island (PAI), a region in the bacterial genome associated with increased virulence. When *H. pylori* infects gastric epithelial cells, CagA is delivered into the host cells via a type IV secretion system. Once inside, CagA undergoes tyrosine phosphorylation and interacts with multiple signaling pathways, causing a range of effects. It disrupts cytoskeletal organization and tight junctions, compromising epithelial barrier integrity. In addition, CagA induces the release of pro-inflammatory cytokines such as IL-1α, IL-8, and IL-18, contributing to chronic inflammation. CagA has also been implicated in the development of gastric cancer due to its ability to interfere with cellular signaling pathways [35]. It inhibits autophagy in host cells, promotes uncontrolled cell proliferation, and suppresses apoptosis. The presence of the CagA gene is associated with more severe clinical outcomes, including peptic ulcers, gastric adenocarcinoma, and a heightened inflammatory response compared to *H. pylori* strains lacking this virulence factor [35]. CagA also promotes the activation of c-Met, which triggers the PI3K/AKT/mTOR signaling pathway. This induces a reduction in autophagy within the host cell and leads to the accumulation of the sequestosome-1 (SQSTM1) protein, further enhancing the production of NF-κB-dependent cytokines [36]. As reported by Xia et al. [37], CagA-positive *H. pylori* strains promote atherosclerosis through exosome-mediated reactive oxygen species (ROS) formation. A study conducted by Rozankovic et al. [38] suggests the existence of autoimmune mechanisms that contribute not only to the pathogenesis of atherosclerotic plaques but also to their destabilization. Other research indicates that CagA antibodies cross-react with antigens present in both normal and atherosclerotic blood vessels. This suggests that the presence of CagA-positive *H. pylori* may influence the progression of atherosclerosis in patients infected with CagA-positive strains [39]. Specifically, cross-reactivity may occur between antibodies targeting lipopolysaccharide-binding protein (LBP) and those directed against *H. pylori* heat shock protein 60 (HSP60), as well as antigens present on endothelial cells and arterial smooth muscle [40]. It has been shown that CagA-positive *H. pylori* strains have an increased ability to stimulate IL-6 production, a cytokine associated with the aging of both vascular and myeloid cells [40,41,42] and induces macrophage cell formation by downregulating the expression of the transcription factors peroxisome proliferator-activated receptor (PPAR)γ and liver-X receptor (LXR)α [43]. Gastric epithelial cells injected with CagA release exosomes containing protein to the systemic circulation, which facilitates the transport of CagA into endothelial cells [44]. In a study conducted on transgenic mice expressing CagA in their endothelial cells, exposure to a high-fat diet induced the development of proatherogenic lesions in the aorta, which were absent in non-transgenic mice exposed to the same diet. These lesions were characterized by an increase in the thickness of the tunica media and a reduction in its elasticity. When the high-fat diet was administered for a longer period, mice with CagA-expressing endothelial cells exhibited greater macrophage infiltration and the development of atherosclerotic plaques [45]. It has also been demonstrated that CagA-positive *H. pylori* strains are associated with increased expression of endothelial adhesion molecules such as ICAM-1 and VCAM-1. These molecules facilitate the binding of circulating monocytes, promoting macrophage infiltration into the endothelium. The upregulation of adhesion molecules is driven by the activation of the NLRP3/Caspase-1/IL-1β pathway, which leads to elevated IL-6 production. This, in turn, promotes local inflammation and, together with macrophage infiltration, contributes to the progression of atherosclerosis [46]. Moreover, it has been demonstrated that the inhibition of exosome secretion with GW4869 effectively prevented excessive aortic ROS production, endothelial dysfunction, and atherosclerosis in mice with CagA-positive *H. pylori* infection [37]. In the meta-analysis published by Shi et al. [47] it has been demonstrated that *H. pylori* can promote atherosclerosis in people under the age of 60 without other cardiovascular risk factors. Recent studies have demonstrated that outer membrane vesicles (OMVs) derived from *H. pylori*-infected gastric epithelial cells encapsulating the CagA are present in the blood of both patients and animal models. This suggests that these OMVs can facilitate the systemic dissemination of CagA into the bloodstream. Building on this evidence, some researchers have hypothesized that *H. pylori* may contribute to the development of atherosclerosis (AS) through OMV-mediated mechanisms [48]. The support this hypothesis it has been shown that the administration of OMVs from CagA-positive *H. pylori* accelerated atherosclerosis plaque formation in ApoE −/− mice [49]. Although these pathophysiological mechanisms have been elucidated and hypothesized, clinical evidence supporting the idea that *H. pylori* eradication improves survival is still lacking. On one hand, this absence of evidence may be attributed to the lack of sufficiently long randomized controlled trials. On the other hand, some authors suggest that the lack of clinical benefit may be due to the dysregulation effects induced by the eradication of antibiotic therapy on the gut microbiota [50]. Figure 1 summarizes the diseases associated with *H. pylori* infection, as well as the effects induced by *H. pylori* that may contribute to the development of coronary artery disease (CAD).

Several clinical studies have highlighted an association between CagA-positive *H. pylori* infection and extra-gastric diseases that share atherosclerotic pathogenesis and risk factors with ischemic heart disease. For example, *H. pylori* DNA has been found in carotid plaques, and CagA-positive strains have shown a higher prevalence in patients with non-cardioembolic stroke, reinforcing this connection [51,52]. A 2019 cross-sectional study and subsequent meta-analyses identified *H. pylori* infection, particularly with CagA-positive strains, as independent risk factors for non-cardioembolic ischemic stroke [53,54]. Collectively, these findings underscore the critical role of *H. pylori* virulence factors, especially CagA, in the development of atherosclerosis and related diseases.

### 2.2. H. pylori and Autoimmunity

Several studies have been led about the link between *H. pylori* infection and autoimmune diseases. Higher serological prevalence rates of *H. pylori* infection have been reported in patients with type 1 diabetes (T1DM) and autoimmune thyroiditis (AT) [55,56]. In particular, Choi YM. et al. [57] found a higher prevalence of *H. pylori* infection in patients with autoimmune thyroid disease and controls. Another study led by El-Eshmawy et al. [58] proved a connection between both type 1 diabetes and autoimmune thyroids, supporting the idea of a connection between *H.pylori* infection and the occurrence of anti- TPO, anti-Tg autoantibodies, and AT in young patients with T1DM. Many autoimmune mechanisms, some of which were described in the previous paragraph, have been considered a potential link to coronary artery disease (CAD). For example, it has been observed that anti-glycan antibodies produced after immunizing animals with heat-killed *H. pylori* strains cross-reacted with histological preparations of infarcted myocardial tissue. Additionally, a cross-reaction between *H. pylori* antibodies and blood group Lewis’s antigens has been documented. These studies suggest that autoimmune phenomena, such as cross-mimicry with *H. pylori*, may play a role in the pathogenesis of CAD by directly promoting thrombotic occlusion through endothelial damage, as well as through local procoagulant phenomena [59].

## 3. *H. pylori* and Predisposition to CAD

Chronic inflammation associated with persistent infections is believed to contribute to the progression of atherosclerotic disease, which may eventually manifest as coronary artery disease (CAD). *H. pylori* presence was detected by polymerase chain reaction (PCR) in 29.5% of 105 patients who underwent coronary artery bypass grafting (CABG) [60]. Additionally, serological evidence of infection was found in 53.3% of these patients. These findings suggest that *H. pylori* infection could play a role in plaque rupture and subsequent ischemic heart disease. Notably, high levels of anti-CagA antibody titers were observed in CAD patients compared to healthy individuals and those with anti-CagA positivity exhibited more severe CAD lesions [61]. Another study evaluated the effects of *H. pylori* eradication, revealing an improvement in CAD. Interestingly, a greater loss of coronary lumen was noted in patients with serological evidence of *H. pylori* infection. However, *H. pylori* eradication attenuated the reduction in coronary artery lumen compared to the placebo group [62]. Further studies are required to explore whether early *H. pylori* eradication may help reduce CAD morbidity.

## 4. Myocardial Infarction

Myocardial infarction (MI) is an acute and severe cardiovascular disease, brought on by ischemia of the heart muscle and blockage of the coronary arteries, poses a significant threat to patients’ lives, and is a serious public health problem [63]. There is a variety of risk factors for MI, such as lifestyle, diet, genetics, and environmental factors [64,65]. Risk factors are divided into modifiable, such as age and ethnicity, and modifiable, such as diet, smoking, and exercise. Reducing these last ones can improve MI prevention and control [66,67,68]. It has been proved that inflammation has a key role in atherosclerosis progression [66]. Inflammation is involved in restenosis or vessel narrowing, after initially successful balloon angioplasty or coronary stenting [69,70]. *H. pylori* gastric infection is one of the common chronic infections and can induce a pro-inflammatory role, which leads to the development of atherosclerosis and the progression of coronary heart disease (CHD) [71,72,73]. A previous study, led by Franceschi et al. demonstrated that anti-CagA IgG can recognize vascular wall self-antigens of 160 and 180 kDa [39], meaning the autoimmune system may also be involved. It is a phenomenon known as “epitope spreading” [74]. In case of an *H. pylori* infection, T lymphocytes activated against CagA positive strains of the bacteria could, therefore, recognize fragments of these self-antigens presented on type I major histocompatibility complex by antigen-presenting cells and stimulate an atherogenic inflammatory response within vascular walls. Moreover, pathogen-induced tissue inflammation may result from local activation of antigen-presenting cells and enhanced processing/presentation of self-antigens that cause T-cell priming, followed by T-cell activation and expansion of additional specificities [75]. A study led by Wang et al. analyzes whether there is a causality of anti-*H. pylori* IgG levels on MI and HDL cholesterol levels. Increased anti-*H. Pylori* IgG levels are significantly associated with an increased risk of MI and decreases in HDL cholesterol levels [76]. A meta-analysis led by Liu J. et al. estimated an approximately 70–100% MI risk increase for *H. pylori* infection [77]. Other studies, not included in this meta-analysis, led in two different areas of Northern Italy and in USA, proved an increased MI risk in patients having a *H. pylori* infection [78,79,80,81]. A study led by Niccoli G. et al. [82] enrolled 181 consecutive patients (155 men, mean age 64 ± 13 years) presenting with STEMI. In all patients, serum levels of IgG anti-CagA were assessed. The results showed that anti-CagA IgG seropositive patients presented more frequently a previous history of acute coronary syndrome compared with seronegative patients. Also, the major adverse cardiovascular event rate was higher in anti-CagA IgG seropositive compared with seronegative patients. These data suggest that CagA-positive strains of *H. pylori* seem to be involved in the pathogenesis of recurring ACS, and CagA seropositivity predicts the outcome of STEMI patients undergoing primary PCI. Table 1 shows the main findings of studies that analyzed the relationship between *H. pylori*, atherosclerosis, and coronary artery disease.

## 5. Methods

This review included papers published from 2000 to 2025 about the relationship between *H. pylori* and CAD. We searched literature reviews, observational studies (case-control, cross-sectional), retrospective and prospective studies, and clinical trials. We selected studies containing data on the association between *H. pylori* infection and CAD, ranging from pathophysiological mechanisms (autoimmune, inflammatory) to clinical aspects. Studies were chosen based on the research period, title, abstract, study type, and English language. We searched on Up-to-Date^®^, PubMed^®^, Web of Science^®^, and Cochrane^®^. This review does not need ethical approval. We included as principal words of research: Helicobacter pylori AND coronary artery disease OR myocardial infarction OR atherosclerosis OR heart; infectious diseases AND coronary artery disease OR atherosclerosis; Helicobacter pylori AND coronary plaques. The authors first selected relevant studies by analyzing the titles, followed by a review of the abstracts. A final selection was made based on a full-text assessment of the remaining papers.

## 6. Discussion and Future Research

The available data seemed to suggest that patients with *H. pylori* infection have an increased risk factor for CAD. Meanwhile, studies are reporting that this association is casual. The relationship between *H. pylori* infection and CAD has been controversial for years. Some studies suggest that the association between *H. pylori* infection and CAD is casual. Studies led on population, have found a higher prevalence of *H. pylori* infection in men than in women, as for CAD. However, it has been shown that *H. pylori* infection is more common in non-Hispanic blacks and Hispanic ethnics and it is linked to poor hygienic conditions. CAD was demonstrated to be prevalent in the Asiatic population and to be linked to psychosocial conditions and factors such as smoking, abdominal obesity, and a raised ApoB/ApoA1 ratio. It has been proven that *H. pylori* infection links to atherothrombosis and brings the organism to a status of chronic inflammation, by stimulating inflammatory mediators, such as in-terleukin-1 (IL-1), interleukin-6 (IL-6), C-reactive protein (CRP), and tumor necrosis factor alpha (TNF-α). This leads to atherosclerotic plaque instability, which is one of the major risk factors for CAD. It has also been proven that *H. pylori* infection worsens hypertension, dyslipidemia, hyper-homocysteinemia, diabetes, and impaired glucose tolerance. These are all factors that lead to a higher risk of CAD. It has been widely proven that there is a relationship between *H. pylori* infection and CAD risk factors, and indirectly to CAD. However, more studies are needed to show whether *H. pylori* infection may be considered a direct risk factor or not and how strongly can be *H. pylori* infection be related to a higher risk of CAD. Whether there could be a strong relationship between *H. pylori* infection and CAD, it would be possible to start screening for *H. pylori* infection as prevention for CAD. This could lead to the use of different treatments, such as antibiotics for atherosclerosis. Such an early screening could also lead to a decrease in CAD risk factors, such as hypertension, dyslipidemia, hyper-homocysteinemia, and diabetes; which are worsened by *H. pylori* infection. This means improved general health for a big slice of the worldwide population. More comprehensive and large studies are required to investigate better this association and to clarify the role of this microorganism in such pathogeneses. This may be helpful to screen for *H. pylori* infection, consider the use of different treatments as antibiotics for atherosclerosis, and improve the lives of many patients with CAD with a strong positive impact all over the world. Future research should include (1) prospective population-based studies in which the incidence or the recurrence of CAD has to be evaluated in correlation with *H. pylori* infection, (2) intervention trials, focusing separately on the chronic and acute phases of CAD; and (3) studies of physiopathology (both in the animal model and humans) to understand the potential biological plausibility.

## 7. Conclusions

Despite the numerous pathophysiological mechanisms underlying the association between *Helicobacter pylori* (*H. pylori*) infection and coronary artery disease (CAD) that have been studied, clinical evidence demonstrating that eradication of the infection provides a tangible clinical benefit is still lacking. Research in this area should be encouraged through prospective studies, as identifying a risk factor as easily addressable as a bacterial infection could represent a crucial step in the prevention of the world’s leading cause of mortality.

## Figures and Tables

**Table 1:** Studies about the relationship among *H. pylori*, atherosclerosis, coronary artery disease and autoimmune disturbances.

Evidence	Studies	Main Findings
*H. pylori* infection and atherosclerosis	*Shi H. Helicobacter. 2022* [47]	*H. pylori* promotes atherosclerosis in people under the age of 60 without cardiovascular risk factors
	*N. Wang. Front. Cell Dev. Biol. 2021* [49]	Outer membrane vesicles secreted by *H. pylori* exert an effect on distant organ and tissue
	*M. Candelli. Int. J. Mol. 2023* [50]	*H. pylori* has a role in the pathogenesis and progress of atherosclerosis
*H. pylori* infection and myocardial infection	*Q. Wang. Front. Microbiol., 2023* [76]	Anti-*H. pylori* IgG levels are associated with an increased risk of MI
	*J. Liu. Helicobacter, 2015* [77]	70–100% increased MI risk for *H. pylori* infected patients
	*R. Pellicano. Int. J. Clin. Lab. 1999* [80]	Increased MI risk in patients with *H. pylori* infection
	*R. Pellicano. Panminerva 1999* [81]	Increased MI risk in patients with *H. pylori* infection
*H. pylori* infection and CAD	*G. Niccoli. Eur. Heart J. ACC, 2017* [82]	In patients with STEMI, Anti-Cag-Ab is associated with history of ACS
	*G. Niccoli. Coron. Artery Dis, 2010* [61]	*H. pylori* is associated with plaque rupture
	*M Kowalski. J. Physiol. Pharmacol. 2001* [62]	*H. pylori* infection was associated with higher loss of coronary lumen
	*NP. Tobin. Am. J. Physiol., 2008* [24]	Eradication of *H. pylori* attenuated the reduction in the coronary artery lumen
*H. pylori* and autoimmune pathologies	*N. Figura et al. Antibiotics 2019* [56]	High prevalence of *H. pylori* infection in patients with Hashimoto thyroiditis
	*M.M. El-Eshmawy. Diabet Met Syn. 2011* [ 58]	*H. pylori* infection is the link between type1 diabetes and thyroiditis

Legend: MI: myocardial infarction, STEMI: ST elevation myocardial infarction, CAD: Coronary artery disease, ACS: acute coronary syndrome.
